# Targeting Tissue Factor to Tumor Vasculature to Induce Tumor Infarction

**DOI:** 10.3390/cancers13112841

**Published:** 2021-06-07

**Authors:** Andrew F. Berdel, Christian Schwöppe, Caroline Brand, Saliha Harrach, Kathrin Brömmel, Heike Hintelmann, Georg Lenz, Ruediger Liersch, Hauke Heinzow, Christoph Schliemann, Rolf M. Mesters, Wolfgang E. Berdel, Torsten Kessler

**Affiliations:** 1 Department of Medicine A (Hematology, Hemostaseology, Oncology, Pneumology), University Hospital Muenster, D-48149 Muenster, Germany; christian.schwoeppe@uni-muenster.de (C.S.); Caroline.Brand@ukmuenster.de (C.B.); s.harrach@gmx.net (S.H.); kathrin.broemmel@ukmuenster.de (K.B.); Heike.Hintelmann@ukmuenster.de (H.H.); georg.lenz@ukmuenster.de (G.L.); liersch@onkologie-muenster.de (R.L.); Christoph.Schliemann@ukmuenster.de (C.S.); Rolf.Mesters@ukmuenster.de (R.M.M.); berdel@uni-muenster.de (W.E.B.); 2 Department of Medicine B (Gastroenterology, Endocrinology, Infectious Diseases), University Hospital Muenster, D-48149 Muenster, Germany; Hauke.Heinzow@ukmuenster.de

**Keywords:** vascular targeting, tissue factor (TF), truncated and retargeted tissue factor tTF-NGR, CD13, aminopeptidase N, tumor vascular occlusion, tumor infarction, solid tumors

## Abstract

**Simple Summary:**

Among multiple other functional roles of tissue factor (TF) and other coagulation proteins in the development and targeting of malignant disease, some scientific groups are attempting to modify TF and target the molecule or truncated forms of the molecule to tumor vasculature to selectively induce local blood vessel thromboembolic occlusion resulting in tumor infarction. This review briefly describes the characteristics and development of some of these proteins and structures, including tTF-NGR, which as the first drug candidate from this class has entered clinical trials in cancer patients.

**Abstract:**

Besides its central functional role in coagulation, TF has been described as being operational in the development of malignancies and is currently being studied as a possible therapeutic tool against cancer. One of the avenues being explored is retargeting TF or its truncated extracellular part (tTF) to the tumor vasculature to induce tumor vessel occlusion and tumor infarction. To this end, multiple structures on tumor vascular wall cells have been studied at which tTF has been aimed via antibodies, derivatives, or as bifunctional fusion protein through targeting peptides. Among these targets were vascular adhesion molecules, oncofetal variants of fibronectin, prostate-specific membrane antigens, vascular endothelial growth factor receptors and co-receptors, integrins, fibroblast activation proteins, NG2 proteoglycan, microthrombus-associated fibrin-fibronectin, and aminopeptidase N. Targeting was also attempted toward cellular membranes within an acidic milieu or toward necrotic tumor areas. tTF-NGR, targeting tTF primarily at aminopeptidase N on angiogenic endothelial cells, was the first drug candidate from this emerging class of coaguligands translated to clinical studies in cancer patients. Upon completion of a phase I study, tTF-NGR entered randomized studies in oncology to test the therapeutic impact of this novel therapeutic modality.

## 1. Brief Introduction on Tissue Factor (TF) in Cancer

Tissue factor (TF) is a 47-kDa membrane-bound cell surface receptor which functions as *primum movens* of the extrinsic pathway in coagulation [1,2]. Among other functions, TF becomes “visible” on the abluminal side of a blood vessel after vessel injury and, positioned by a transmembrane domain on the cellular surface, forms a complex TF:factor VIIa:factor X (TF:FVIIa:FX), which in the presence of phospholipids activates FX to FXa and induces a coagulation cascade. In this major function of the molecule, TF plays a role in various states of different diseases, among them cancer [3,4]. As first observed by Trousseau in the 19th century, cancer patients are characterized by a comparatively high thromboembolic risk. This risk can even be quantified in a clinically useful way, helping to reduce the burden and consequences of venous thromboembolism in patients with cancer by consequent thromboprophylaxis [5]. As thromboembolism is indeed a cause of death in cancer patients receiving chemotherapy [6], standards of anticoagulation in these patients are fine-tuned by numerous clinical studies. In some of these studies circulating TF was proposed as a biomarker for recurrent venous thromboembolism [7].

### 1.1. TF and Tumor Biology beyond Coagulation

Connected with its role in coagulation and thromboembolism, but also far beyond, TF biology in cancer is complex [8,9]. The molecule has been connected with tumor biology, growth and metastasis in multiple ways [10,11], some of which are discussed in other papers in this Special Issue of Cancers. Among these important aspects, some are worth to be briefly mentioned here: Induced by epithelial-mesenchymal transition (EMT) in circulating tumor cells, TF can promote metastasis by a procoagulant state of these cells [12]. Independent from its procoagulatory function, TF was also described to be prometastatic [13], and the inhibition of TF signaling can suppress tumor growth [14]. The molecule is operational in tumor blood vessel formation [9]. Further, signaling of the TF:factor VIIa:factor Xa (TF:FVIIa:FXa) complex regulates innate toll-like receptor dependent interferon response and tumor immune-evasive chemokine expression [15]. The TF:FVIIa ligand FX has been described to dampen antitumor immunity, which might be even therapeutically exploitable by targeting the FX-protease-activated receptor 2 pathway with FXa antagonists [16]. Thus, in the complex interactions between tumors, tumor host tissue and TF the molecule acts in a rather tumor-promoting way.

### 1.2. TF as a Target for Tumor Therapy

Since many tumor entities have been described as being TF-positive in immunohistochemistry and the presence of higher amounts of TF in tumors correlates with worse prognosis [9,10,17,18], tumor TF has been chosen as a target for experimental cancer therapies. To this end, anti-TF antibodies with different payloads including toxins and radioactive ligands or FVII peptide immunoconjugates have been studied [19,20,21]. One group has attempted to target tissue factor on angiogenic endothelial cells and tumor cells with FVII-targeted photodynamic therapy, with tumor vascular collapse as one potential result [22,23]. Finally, a toxin anti-TF antibody conjugate, tisotumab-vedotin, is in early clinical trials [24].

## 2. Re-Targeting TF to Tumors as Experimental Cancer Therapy

In an attempt opposite to what has been described so far, several groups including our laboratory have tried to target TF or procoagulatory parts of the molecule at the tumor vasculature to selectively induce tumor vessel occlusion with resulting tumor infarction, tumor shrinkage, or inhibition of further tumor growth and spread. In general, this field was kicked off by the ground-breaking hypothesis of J. Folkman in 1971 [25] that tumor neo-angiogenesis could lead to anti-angiogenic tumor therapy, and a decade later more specifically by J. Denekamp [26,27], who hypothesized that proliferating tumor endothelial cells should carry specific markers, which could be exploited for targeting of anti-tumor maneuvers. In the following, the approach of re-targeting TF is reviewed in more detail, going by the tumor targets used for re-targeting the molecule as a primary order for subheadings rather than by chronological order of publication.

### 2.1. Major Histocompatibility Complex Class II (MHC II) Antigens

P. Thorpe’s laboratory was the first to propose a strategy of targeting the extracellular part of TF to a marker specific to the tumor endothelium [28]. At that time naturally occurring mouse tumor endothelial markers were not described in detail. Thus, they used a mouse model, in which the tumor vascular endothelial cells specifically expressed an MHC II marker. This expression was induced by transfection of the transplanted mouse neuroblastoma cell line C1300(Muγ) with the murine interferon gamma gene. To target the truncated TF (tTF), lacking the transmembrane domain of the molecule, they used a bi-specific antibody with one Fab arm binding to MHC II, and the other to the C-module of tTF. This antibody mediated binding of tTF to the tumor endothelial cell surface in an antigen (MHC II) specific manner. Mechanistic studies reported tumor vascular thrombosis and a resulting inhibition of neuroblastoma growth in vivo in mice treated intravenously (i.v.) with these constructs. Early after the i.v. injection of these newly termed “coaguligands”, tumors became “bruised and blackened” and later “collapsed” [28].

### 2.2. Vascular Adhesion Molecule-1 (VCAM-1, CD106)

Thorpe’s group subsequently followed with a study using VCAM-1 as a target [29]. VCAM-1 was the first marker which they were able to target as a naturally occurring molecule present on the cell surface of tumor vascular endothelium. They used the human Hodgkin lymphoma cell line L540 xenotransplant model for treatment experiments. In contrast to their previous report [28], in this study instead of designing a bispecific antibody linking tTF to the target, an anti-VCAM-1 antibody was directly linked to tTF (VCAM-1-tTF). This coaguligand was active as therapeutic agent since it caused tumor vessel thrombosis and tumor growth retardation. However, a note of caution was that VCAM-1-tTF also localized to VCAM-1 positive vessels in heart and lung tissue, although no thrombosis occurred in these blood vessels. In this report [29], the authors described selective expression of phosphatidylserine (PS) in tumor versus lung and heart vasculature cells as a possible reason for this functional difference, since PS presence on the surface is important for TF to start the extrinsic coagulation pathway. Although these studies were encouraging, the group did not further develop this approach. Instead, a company founded by some of the authors, Peregrine, concentrated on the development of an anti-PS antibody bavituximab for cancer therapy, which obtained fast track designation by the FDA, but unfortunately failed in late-stage development.

The VCAM-1 targeting of TF, initially started in P. Thorpe’s laboratory [30], was studied in further human tumor xenograft models by C. Gottstein’s group in Cologne [31]. A fusion protein consisting of an anti-CD106 antibody linked to soluble (s) TF (α-hCD106-sTF) was effective in retarding the xenotransplant growth of human L540rec Hodgkin lymphoma cells, and two subtypes of Colo677 (first thought to be of small-cell lung cancer origin, later recategorized). The binding of α-hCD106-sTF to endothelial cells in vitro, FX activation in vitro, and tumor vascular thrombosis and tumor necrosis in histology could be shown as the mechanistic basis of the therapeutic effects. The therapeutic activity in vivo could be further increased by addition of lipopolysaccharide to α-hCD106-sTF.

### 2.3. ED-B Domain of Fibronectin

The antibody technology described above was modified by D. Neri’s group. On the basis of a broad laboratory program designing and testing immunocytokines to target a growing number of tumor vascular markers described [32], the authors tested selective thrombosis of tumor blood vessels by designing a fusion protein consisting of an scFv fragment of an antibody against the oncofetal ED-B domain of fibronectin (L19) coupled to the N-terminus of tTF. ED-B is selectively expressed along the vasculature of actively remodeling tissues such as tumors, but not in normal tissues (with few exceptions). Its domain sequence is identical between mice and humans. The fusion protein L19-tTF was described to mediate selective infarction of 3 different solid murine tumor models including complete tumor eradication [33].

### 2.4. Prostate-Specific Membrane Antigen (PSMA)

These observations were then further followed by T.S. Edgington’s laboratory, who used PSMA as a target [34]. PSMA is a carboxypeptidase antigen expressed on prostate and other carcinoma cells and also on tumor microvascular lining cells. The authors stated that tumor cells lining a vasculature-like structure in the tumors, a process named “vasculogenic mimicry”, also expressed PSMA. The extracellular tTF was coupled to a PSMA catalytic site inhibitor. This streptavidin-tTF (Strep-TF) molecule had the targeting moiety on the N-terminus of tTF. Strep-TF induced tumor infarction in the human LuCap prostate cancer murine xenograft model and in a rat Mat Lu prostate tumor in vivo. Of note, a large dose range of Strep-TF in rats was tolerated well. Tumor growth inhibition by systemic PSMA-directed Strep-TF could be rendered considerably more effective by combination with low dose liposomal doxorubicin. Whereas therapy with Strep-TF alone had a significant but only minor tumor growth retardation as a consequence, tumor growth was stopped completely over an observation period of 16 days by the combination.

### 2.5. Vascular Endothelial Growth Factor Receptors (VEGFR) and Co-Receptors

Within a search for novel tumor vascular targets T.S. Edgington’s laboratory next concentrated on the vascular endothelial growth factor (VEGF) isoform containing a heparin-binding domain (HBDt) as a potential target [35]. They had shown before that this domain was localizing on surfaces of tumor endothelial cells. Thus, a fusion protein named HBDt-TFt, in which HBDt was fused to the N-terminus of the extracellular part of TF with a short spacer in between, was studied. After systemic application to tumor-bearing mice, selective and occlusive thrombosis in the tumor microvasculature occurred, rapidly followed by tumor infarction. Combination of HBDt-TFt with FVIIa even resulted in tumor eradication. A trimolecular complex consisting of chondroitin C sulfate proteoglycan, neuropilin-1, and VEGF receptor-2 (VEGFR2, KDR) overexpressed together in angiogenic sites of the tumors was microscopically determined to be the actual binding site for HBDt-TFt.

Later, a similar fusion, in which the peptide SP5.2 with a sequence of 16 amino acids selectively binding the VEGFR1, was N-terminally linked to tTF by X. Li’s group at the Xiamen University in Fujian, China. This construct was functionally studied in vitro for binding to human umbilical vein endothelial cells (HUVECs) and retaining procoagulatory activity. In vivo it showed tumor homing upon systemic application of a fluorescent derivative and inhibitory effects in a sarcoma mouse model [36].

Neuropilin-1 (NRP-1) is a co-receptor for VEGF. Recently, the same university in Fujian, China, chemically coupled an anti-NRP-1 antibody into an anti-NRP-1-streptavidin:tTF-biotin “composite system” and obtained similar tumor vascular thrombosis and tumor growth inhibition effects [37]. The authors argued that the streptavidin and biotin components of the system enhanced thrombogenic activity. The same group later constructed a recombinant protein consisting of tTF and the NRP-1 targeting peptide EG3287 in the C-terminus of tTF [38]. tTF-EG3287 targeted to tumors was reported to selectively activate coagulation in tumor vessels upon target binding, and to inhibit tumor growth of a HepG2 tumor model in mice without visible side effects such as thrombosis in normal organs [38].

### 2.6. Integrins

R. Pasqualini in E. Ruoshlathi’s group described organ targeting in vivo by phage display of peptide library techniques [39] and later loaded promising peptides with different active payloads for targeting. They detected peptides binding to integrins, namely αvβ1, αvβ3, and αvβ5, which are preferentially present on tumor endothelial cells [40,41,42,43]. A. Epstein’s group subsequently generated tTF fusion proteins with one of these peptides (CDCRGDCFC) carrying a tripeptide RGD (arginine-glycin-aspartate) motif on the N-terminus, RGD/tTF, and tested this for induction of tumor vessel thrombosis [44]. This fusion protein only induced thrombosis in capillaries and small tumor vessels, an effect which was insufficient to inhibit tumor growth in vivo. In contrast to RGD/tTF, two other fusion proteins generated and comparatively tested induced thrombosis in small and medium tumor vessels and significantly retarded growth of a MAD109 tumor in BALB/c mice in vivo. Both were constructs of antibodies positioned to tTF. One, chTNT-3/tTF, targeted necrotic regions of a tumor in which conserved and abundant intracellular antigens are exposed [45,46], the other, chTV-1/tTF, targeting fibronectin, which is located in the basement membrane of vessels accessible in fenestrated (leaky) tumor endothelium [47].

Despite the disappointing results with RGD/tTF, we kept our parallel project active, in which we generated a fusion protein consisting of tTF and an RGD motif, GRGDSP, positioned to the C-terminus of tTF [48]. This fusion protein, tTF-RGD, showed RGD-specific binding to αvβ3 integrins and serum-stimulated endothelial cells, massive tumor vessel occlusion, blood pooling and extravasation in tumor histology, as well as growth retardation upon systemic application in murine xenograft models of human lung cancer, melanoma and sarcoma xenografts in vivo. In contrast, lack of off-target thromboembolic events in vital normal organs, such as lungs, heart, kidneys and liver of the tumor-bearing mice at therapeutically active doses suggested a therapeutic window.

In contrast to the negative results of RGD/tTF, a model fusion protein in which the targeting peptide was bound to the N-terminus of tTF, the COOH-terminal positioning of the targeting RGD-moiety in our tTF-RGD was unexpectedly successful. We had theoretically deduced this conformation by replacement of the transmembrane peptide present in the identical C-terminus position of the naturally occurring TF, which renders the molecule procoagulative by forming the TF:FVIIa complex and activating FX on the cell membrane in the neighbourhood of PS-rich phospholipids. When considering the crystal structure of the TF:FVIIa complex [49], linkage of the RGD peptide to the N-terminus of tTF would cause an orientation that is opposite to the orientation of native TF.

### 2.7. Fibroblast Activation Protein (FAP)

In 2000 Rippmann et al. reported on a fusion protein composed of a humanized scFv targeting FAP, abundantly present in tumor tissues and the extracellular domain of TF [50]. This construct TFOS4 was functionally described as procoagulatory upon binding to FAP-positive cells, but it was not further developed for experimental cancer treatment.

### 2.8. NG2 Proteoglycan on Angiogenic Pericytes

Within our search for better targets directing tTF via peptides linked to the C-terminus of the molecule into the tumor vasculature, we have also tested NG2 as a surface antigen on angiogenic pericytes [51]. NG2 is expressed on the abluminal side of the endothelium and could be reached through the leaky tumor vasculature. However, although the fusion proteins tTF-TAA and tTF-LTL clearly maintained the procoagulatory activity of the tTF moiety, the binding affinities to the pericyte targets were rather low with a dissociation K_D_ in the higher nanomolar range, and the in vivo therapeutic effects against human tumor xenografts were disappointing with a rather small therapeutic window.

### 2.9. Microthrombus-Associated Fibrin-Fibronectin Complexes

In vivo screening of phage-displayed peptide libraries for tumor homing in tumor-bearing MMTV-PyMT transgenic breast cancer mice by Ruoslahti’s group also identified a pentapeptide, CREKA [52]. As CREKA revealed binding to fibrin and fibrin-associated clotted plasma proteins and subsequently self-amplified targeting and binding by new binding sites preferentially inside tumors, the laboratory used this peptide to accumulate nanoparticles coated with CREKA in the tumor vasculature [52]. Later, this peptide in a gadolinium-based contrast agent (CREKA-Tris (Gd-DOTA)_3_) was described as binding to fibronectin-fibrin microthrombi inside tumors undergoing EMT, and thus was used for magnetic resonance imaging (MRI) detection of 4T1 breast cancer micrometastasis models [53].

Recently, G. Nie’s group in Beijing described a tTF-CREKA fusion protein as procoagulatory, accumulating in tumors in biodistribution studies, and inducing tumor-selective intravascular thrombosis. tTF-CREKA inhibited growth in model tumors of breast (4T1), hepatocellular (MHCC97H) and colon (LS174T) origin [54].

### 2.10. Acidic Tumor Endothelium

In the series of antitumor tTF-constructs made and tested by G. Nie’s group, in some model tumors tTF-CREKA was more efficient than a fusion protein, tTF-pHLIP, a construct forming a helical structure inserting into cellular membranes at low pH [55]. However, this construct also was also described as being therapeutically active in different tumor models and well tolerated by the animals at therapeutically active doses. A further conceptual attraction the authors discussed was that tTF-pHLIP does not need a tumor endothelium-specific ligand–receptor interaction but exploits the acidic milieu inside tumors. However, whether this led to a more tumor specific vascular accumulation or a larger therapeutic window was not comparatively studied.

## 3. Re-Targeting TF to Tumors—Non-Clinical Results and Translation into the Clinic

### 3.1. CD13, Aminopeptidase N

It was again R. Pasqualini’s laboratory group who proposed the binding of small NGR (asparagine-glycine-arginine)-containing peptides to aminopeptidase N (APN, also known as CD13) for tumor vascular targeting [56]. Later, during the process in which our group defined tTF-NGR as being a lead compound for further development of this treatment modality, CD13 [57,58] was shown to promote angiogenesis, tumor growth, and metastasis [59]. CD13 was later reported to be of prognostic relevance for patients with cancer of some but not all histologies [60,61,62,63,64,65,66].

In 1998, stimulated by P. Thorpe’s work [28], our laboratory started to design a group of new bifunctional tTF fusion proteins to target procoagulatory activity selectively into the tumor vasculature and to induce tumor infarction [48,51,63,65,66,67,68,69,70,71,72,73,74,75,76,77,78,79,80,81].

#### 3.1.1. Non-Clinical Pharmacodynamic Data with tTF-NGR

**Figure 1:**
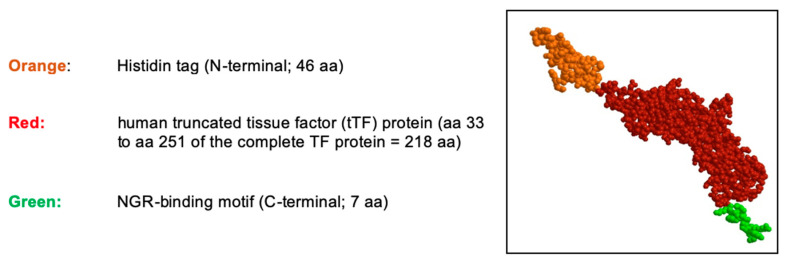
Schematic structure of the tTF-NGR protein. The truncated tissue factor (tTF) in red, the C-terminal NGR-peptide binding motif in green, and the N-terminal Histidin tag (His tag) in orange. The molecular weight is 30,381.98 dalton, the NGR motif is located on the C-terminus of tTF. aa, amino acids.

During these studies, his-tag-tTF-GNGRAHA (tTF-NGR) evolved as the lead compound due to most stable antitumor activity. The fusion protein is graphically depicted in Figure 1.

tTF-NGR retained the procoagulatory activity of TF in a FX/FXa assay [69,70,71] and it revealed specific binding to the respective target molecules (CD13) on stimulated endothelial cells in vitro [70,71,72] and to tumor endothelium in vivo [65]. Multiple imaging techniques showed in vivo accumulation of tTF-NGR in tumor tissues and its in vivo-induced activation of coagulation with subsequent tumor vascular occlusion and inhibition of tumor vessel blood flow upon systemic application [67,68,69,70,71,72,75,80]. In contrast, these central activities were not observed when using tTF without a targeting moiety. The tTF-NGR protein was then tested against a broad range of human tumor xenografts from different histological origins growing in athymic mice and also against syngeneic mouse tumor models. The molecule revealed therapeutic efficacy, leading to the inhibition of tumor growth and in some instances the shrinkage of established tumors, again in contrast to tTF without targeting moiety. A summary of the results is depicted in Table 1.

We have performed orienting dose-response studies in our early xenograft mouse experiments. To this end, we have repeatedly found single doses of 0.5 mg/kg body weight to be ineffective for tumor growth inhibition and 0.75 mg/kg as a dose with marginal activity. These early studies were done also in the HT1080 fibrosarcoma model and the results are among those given in Table 1 under the experiments without a therapeutic effect. Later, repeated intravenous injection of at least 1 mg tTF-NGR/kg body weight was a standard therapeutical dose in xenograft experiments.

**Figure 2:**
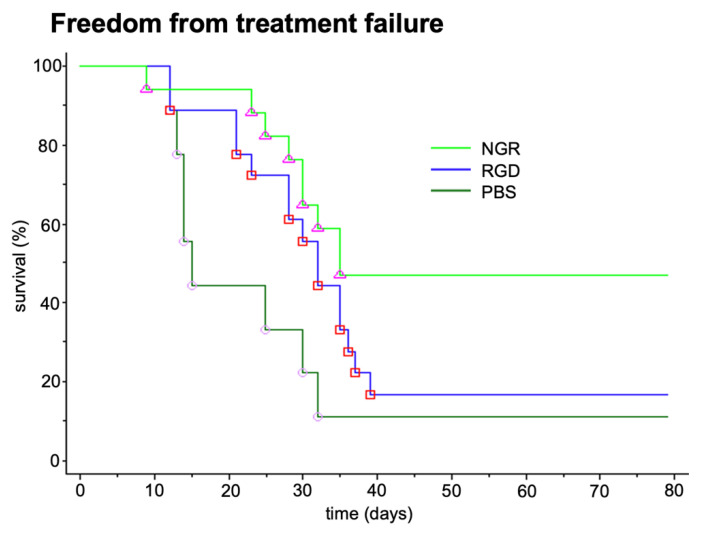
Antimetastatic effect of tTF-fusion proteins tTF-NGR (NGR) and tTF-RGD (RGD). Antimetastatic effect of two tTF-fusion proteins systemically given at 3 mg/kg body weight every 2–3 days x5 in a syngeneic B-16 melanoma model growing in C57BL6 mice. Freedom from treatment failure: spontaneous metastasis after surgical removal of the subcutaneous primary tumors was clinically observed (e.g., inactivity, dyspnea, weight loss, fur changes), subsequently the mice were sacrificed (survival time) and the lungs of the animals were examined for lung metastasis. When compared with saline controls tTF-NGR revealed significant superiority (Mann-Whitney test, *p* = 0.009). PBS, saline control. Unpublished data.

In addition to the tumor growth inhibition in the xenograft models of human tumors, an antimetastatic effect was seen in a syngeneic B-16 melanoma model when targeted tTF was given close to the surgical removal of the primary tumor (Figure 2).

As locoregional toxicity leading to necrosis of mouse tail tips after repeated intravenous injections of the concentrated tTF-NGR occurred, the molecule was polyethylene glycol conjugated (PEGylated) in a random [76] modification at multiple sites of the molecule and in a site-specific [77] modification. PEGylated tTF-NGR showed better local and also systemic tolerability, however, the therapeutic window did not broaden up in a way justifying the more complex good manufacturing practice (GMP) construction protocol.

**Figure 3:**
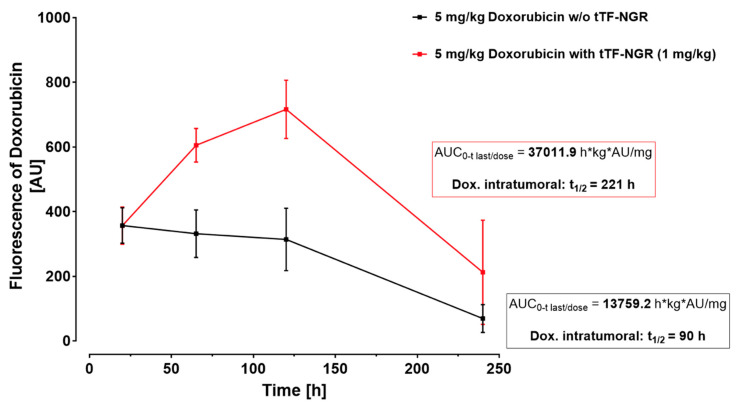
Intratumoral doxorubicin concentration with or without subsequent vascular occlusion by systemic tTF-NGR. Fluorescence-based semi-quantification of doxorubicin tumor accumulation and wash-out kinetics without tTF-NGR (black line) versus with tTF-NGR (red line) applied 6 h after doxorubicin for the first time. The combination protocol significantly retarded wash-out times of doxorubicin from the tumor with prolonged high intratumoral drug levels in the tumor tissue after 65 and 120 h upon doxorubicin-tTF-NGR sequences as compared to control sequences of doxorubicin-saline (*p* = 0.0043 at 65 h, *p* = 0.0095 at 120 h, Mann–Whitney test). AU, arbitrary units; AUC, area under the curve; kg, kilogram body weight; t_1/2_, tumor elimination half-life. For methodological details see Ref. [79].

Interestingly, therapeutic combination protocols using tTF-NGR in specific sequences with regional ultrasound [74], radiotherapy [81] or cytotoxic compounds such as doxorubicin [79] considerably increased the therapeutic efficacy of the molecule. Whereas in the monotherapy mostly retardation of tumor growth was observed, combinations yielded regression of established tumors or completely inhibited tumor growth. The mode of action leading to the combination effects with radiotherapy was shown as being based on an enhanced procoagulatory activity of tTF-NGR bound to the irradiated tumor endothelial cells after expression of higher concentrations of phosphatidylserine (PS) on their outer cell surface following pro-apoptotic effects of the radiation [74,81]. Similar effects could also be shown to increase the procoagulatory effects of tTF-NGR upon exposure of tumor vasculature cells to the apoptosis-inducing activity of doxorubicin [79]. In addition, the combination of first administering doxorubicin followed by subsequent tumor vascular occlusion by tTF-NGR at high intratumoral drug levels increased the intratumoral doxorubicin concentrations and the duration of tumor cell exposure to the drug (Figure 3) [79]. This was in contrast to what was observed in normal organs. Thus, this combination revealed a two-sided enhancement of the therapeutic effects of the single combination partners [79].

#### 3.1.2. Translational Considerations and Non-Clinical Safety and Pharmacokinetic Data for tTF-NGR

Whereas TF or procoagulatory parts of the molecule retargeted to tumors with different molecular constructs and within multiple studies in the last 20 years have shown promising anti-tumor activity in various preclinical models, none of the molecules studied have been consequently developed into an investigational new product (IMP) for clinical application in cancer patients. Certainly, there is a medical need to improve the outcome of different advanced cancer entities by novel treatment modalities. Among other reasons for this hesitation are concerns about off-target procoagulatory effects of tTF not bound to the targets on tumor vasculature cells leading to systemic thromboembolism. To this end, it is important to note that soluble TF without the transmembrane domain was reported to have minor procoagulatory effects, but its activity is restored once TF is anchored into a phospholipid PS rich cellular membrane [82,83]. On the other hand, soluble TF can retain some thrombogenicity, e.g., by promoting the growth of preexisting thrombi [84]. However, with increasing concentrations applied off-target procoagulatory effects are possible and even on-target effects, for example, tTF-RGD binding to integrins present on platelets, could lead to systemic thromboembolism.

tTF-NGR primarily binds to the aminopeptidase CD13. In contrast to regular mature vascular tissue, CD13 is predominantly prevalent in juvenile and growing endothelial tissue and is therefore mainly found in the endothelial cells (EC) of tumor vasculature [85]. Additionally, CD13-targeted tTF is dependent on the coagulation-competent environment of blood vasculature for it’s mechanism of action, and therefore is inactive in other tissues expressing CD13, for example in small bile ducts. Thus, selectivity for tumor vasculature and thereby cancer of the activity of these fusion proteins is based on coupling functional environments with anatomical structures. On this basis we decided to develop this molecule further for clinical studies in cancer patients.

After setting up a GMP process and laboratory using *E. coli* fermenters and a 4-step purification process on HPLC as described in detail [71], and obtaining a manufacturer’s license, preparation for a clinical phase I study was started. Animal safety and toxicology studies in accordance with ICH M3 and S6 (as modified in S9) guidelines of the European Medicines Agencies (EMA) were conducted in four species, including non-rodents, to select a safe starting dose. In addition, the pharmacokinetics of the molecule were established in beagle dogs. The results of these studies have been recently published [86]. In addition to these non-clinical safety studies, we had previously treated a few individual patients with advanced cancer beyond all standard therapy options with low doses of tTF-NGR [70], and these data in retrospect were also consulted for establishing a safe starting dose for phase I.

#### 3.1.3. Clinical Data—Phase I Trial with tTF-NGR in Cancer Patients

tTF-NGR has recently been investigated in a first-in-class phase I study, which recruited a total of 17 patients suffering from a variety of different solid tumor entities with previously exhausted standard therapy [87,88]. The study protocol was approved by the Ethical Board of the Physicians’ Chamber of Westphalia-Lippe and the Westphalian Wilhelms University of Muenster, Germany (AZ 2016-414-f-A) and by the Paul Ehrlich Institute (PEI), Langen, Germany. The study was conducted in accordance with the Declaration of Helsinki (version: Fortaleza, Brazil, 2013) and written informed consent by the patients was obligatory prior to entry to the study.

A daily application of tTF-NGR for 5 consecutive days (1-h infusion, central venous access) with rest periods of 2 weeks between cycles was chosen, and the starting dose was 1 mg/m^2^ body surface area (bsa) per day. The protocol allowed for intraindividual dose escalation and once dose-limiting toxicity (DLT) was observed, maximum tolerated dose (MTD) was established in verification cohorts of 6 patients. The primary objectives of the study were safety and tolerability including establishing DLT and MTD for the protocol applied. Secondary objectives were to assess for tumor blood flow reduction under therapy as proof of principle, tumor response, and to perform pharmacokinetic studies of tTF-NGR. MTD was reached at 3 mg/m^2^ tTF-NGR/day × 5, q day 22. DLT was observed as an isolated significant increase of high sensitivity (hs) Troponin T hs. This increase remained without clinical sequelae and was completely reversible upon discontinuation of treatment. Three thromboembolic events (grade 2), tTF-NGR-related besides other relevant risk factors, were observed within this group of 17 patients treated. The dose levels were 3, 4, and 5 mg/m^2^ bsa (1 event at each level) respectively, and although the numbers were small, a dose-related occurrence is possible. All 3 thromboembolic events were completely reversible upon anticoagulation and the ceasing of further infusions. 

**Figure 4:**
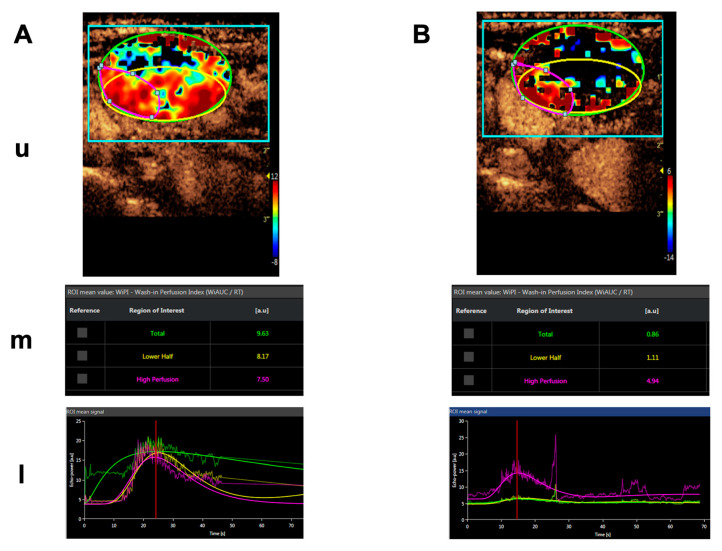
Contrast-enhanced ultrasound (CEUS) imaging series of a lymphnode metastasis of a medullary thyroid carcinoma. (**A**). Shortly before treatment. (**B**). After 5 days of daily treatment with 4 mg/m^2^ bsa tTF-NGR. Upper panels for (**A**,**B**) (**u**): Heat maps of dynamic CEUS. Color scales at the right lower corner of each photograph semiquantify contrast perfusion (red = high, blue/black = low). The green circles represent “regions of interest” (ROI) for the “total” lesion, the yellow circles represent the “lower half” of the lesion, and the red circles represent a “high perfusion” area of the lesion. Middle panels for (**A**,**B**) (**m**): CEUS arbitrary units (a.u.) for the wash-in perfusion index (WIAUC/RT; for the methodological details see [88]) representing the contrast flow through the vasculature of the lesion. For color representation of the 3 different ROI measured, see upper panel. Lower panels for (**A**,**B**) (**l**): Graphic follow-up of the wash-in perfusion index representing the contrast flow through the vasculature of the lesion for 70 sec starting with the injection of contrast via peak-enhancement until wash-out. For color representation of the 3 different “regions of interest” (ROI) see upper and middle panels.

Importantly, blood flow was significantly and specifically reduced in the tumor tissue of all measurable lesions as shown by dynamic contrast-enhanced ultrasound (CEUS) and dynamic contrast-enhanced (DCE) magnetic resonance imaging (MRI), serving as evidence for the mechanism of action of tTF-NGR. An example of a CEUS imaging series in one patient is shown in Figure 4. Induced by therapy with tTF-NGR, contrast flow through the vasculature of the metastatic lesion decreased by more than 1-log step in the total lesion. However, the blood flow reduction was less pronounced in a “high perfusion” area and in parts of the rim of the lesion. Normal liver tissue did not show this blood flow decrease. Following tTF-NGR application some tumor lesions developed intratumoral hemorrhage and necrosis, although no definitive treatment response was reached according to the Response Evaluation Criteria in Solid Tumors (RECIST). At 8 to 9 h the terminal half-life of tTF-NGR was reached without accumulation following daily infusions. In summary, the single-agent treatment regimen with tTF-NGR was proven to be safe for administration, and imaging correlation of significant reduction of blood flow in tumor lesions served as evidence for the mechanism of action.

## 4. Discussion

The last two decades have seen multiple experimental projects incorporating TF into bifunctional proteins and retargeting the molecule to the cells of the tumor vasculature wall for induction of tumor infarction. With some structural exceptions [44,51], the published reports have successfully shown the thrombogenic efficacy of the constructs, tumor targeting, tumor vessel occlusion and tumor growth inhibition with a therapeutic window consistent with safety and tolerability. The common denominator starting most of the published studies was not the functionality of TF within these constructs, but rather the search for an ideal target in the tumor vasculature. Although modern technologies such as gene expression arrays or RNAseq might deliver better tumor or even tumor vasculature targets in the future, reviewing the multiple targets attempted for accumulating TF into tumor vessels, there was no ideal target candidate when we started to select a lead protein for translating targeted TF into the clinic. Limitations concerning biodistribution, low affinity of the targeting moiety inside tumors lacking complete vessel occlusion, off-target undesirable side effects such as systemic thromboembolism, potential immunogenicity of the fusion proteins and fast degradation by the reticulo-histiocytic system could always be discussed.

However, with the reproducibility of the efficacy and safety data in animal models and the urgent medical need to improve therapy for advanced cancer by new treatment modalities, our group decided to translate the drug candidate tTF-NGR into clinical testing in cancer patients.

The primary target for tTF-NGR binding to tumor vascular cells is aminopeptidase N (APN), classified as CD13 [58]. CD13 is a transmembrane enzyme present on cells of different tissues with multiple functions, with some being important for tumor and tumor vasculature invasiveness [56,57,58,59,60]. Supporting the choice of this target were the following facts: CD13 is preferentially present and overexpressed on tumor vascular wall cells, primarily on tumor endothelial cells but also on pericytes that take part in angiogenesis, and CD13 is essential for capillary tube formation [58,89,90]. There are other NGR-targeted drugs in clinical studies of phase II and III in oncology such as NGR-targeted tumor necrotizing factor NGR-hTNF [91,92,93]. CD13 was already shown to be a clinically useful and meaningful target in studies with other molecules, e.g., in a randomized study with bestatin [94,95]. The molecule has also been successfully used as target for experimental tumor imaging with various labeled NGR-constructs [96,97,98,99,100]. The safety profile in phase I with tTF-NGR compares favorably to a variety of other anticancer drugs. In particular, the increase of Troponin T hs potentially is a sensitive and early biomarker for safety [88]. Furthermore, the thromboembolic events observed at higher doses were only grade II and completely reversible upon drug withdrawal and anticoagulation, these kinds of specific and prompt countermeasures not always being available for other drugs approved for clinical use in oncology. On the other hand, caution is necessary: CD13 is also present on some tissues of normal adult organisms [85], and the notion that the binding of NGR-peptides is highly specific for a CD13-isoform exclusively overexpressed on tumor vessels [57] has only been shown for the CNGRCG-peptide in NGR-hTNF, but not for GNGRAHA in tTF-NGR. Thus, areas of vascular remodeling, such as in ischemic myocardium, wound healing, or periodically in the uterine wall, in the placenta in pregnancy, and in small vessels that sometimes express CD13 such as in the CNS [85], or areas with pre-existing microthrombi, are a theoretical concern for the application of tTF-NGR and define exclusion criteria for the use of tTF-NGR. Further, NGR peptides undergo slow and spontaneous deamidation to L-isoaspartate-glycine-arginine (isoDGR) [101,102] and by this process generate a ligand to alpha v integrins also upregulated on tumor vessel wall cells [41,101,103]. Although it might be of theoretical advantage to target tTF-NGR to two intratumoral binding sites, to have a clear and predictable mode of action, we have excluded this phenomenon by modifying the GMP process, storage and use of the molecule. Interestingly, A. Corti’s group has shown that some NGR-peptide degradation and post-translational modifications can be reduced by the introduction of an N-terminal serine [104].

To shed some more light on the field, similar, but also methodologically separate studies have attempted to increase intratumoral coagulation without targeting a specific molecule within the neoplastic tissue via a bifunctional fusion protein. To this end, it is interesting that soluble TF when coadministered with low-dose lipopolysaccharides has been described as inducing coagulation on tumor endothelial cells [105], an effect that could even be enhanced by radiosurgery as shown in a glioblastoma model [106]. Rapamycin was described as inducing tumor thrombosis via TF in the presence of VEGF [107]. S. Bhatia’s group using tTF-RGD and tTF-NGR has described nanoparticles that communicate in vivo to amplify tumor targeting and intratumoral coagulation and intratumoral doses of chemotherapeutics [73]. G. Nie’s group has constructed nanoscale robots [108,109] triggering the intratumoral release of thrombin or thrombin and cytotoxic molecules.

In addition, Seidi et al. have used the GNGRAHA peptide for targeting molecules than other TF, e.g., staphylococcus coagulase in tumor vessels, and observed tumor accumulation and infarction in a PC3 human prostate tumor model with tolerability at therapeutic doses [110].

Meanwhile, as phase I data are available, tTF-NGR has entered randomized clinical trials in cancer patients, one of which will compare progression free survival of patients suffering from advanced soft tissue sarcomas treated in 2nd line with trabectedin versus trabectedin plus tTF-NGR (EudraCT-Nr: 2020-005858-21). We hope these randomized trials will give conclusive data on the clinical impact of the first drug candidate for this novel treatment modality. Only then can we answer the question of whether one of the approaches described can reveal an “upside of coagulation in cancer” [111] and can be therapeutically exploited in oncology.

## 5. Conclusions

The last two decades have seen multiple experimental projects to incorporate TF into bifunctional proteins and retarget the molecule to tumor vasculature wall cells for inducting vascular occlusion and tumor infarction. This has been successfully exploited in different experimental models for cancer therapy. A first drug candidate, tTF-NGR, was translated into the clinic and has shown applicability in a phase I trial. It has now entered a randomized study in patients with metastasized sarcoma. However, due to potential side effects, further studies with tTF-NGR must be performed with caution and in a clinical setting with specific hemostaseology expertise available.

## 6. Patents

W.E.B. and R.M.M. are inventors of patents on vascular targeting with tissue factor-constructs.

## Figures and Tables

**Table 1:** List of in vivo studies investigating the antitumor efficacy of tTF-NGR in tumor models of various histological origin (collected data from tTF-NGR Investigator’s Brochure).

**Xenograft Model** **(Cell Line/** **Mouse Strain nu/nu)**	**Histology** **(Human)**	**No. of Experim.** **with ≥ 5 Mice/Group**	**Therapeutic** **Effect ^a^ (+/−)**	**Imaging** **Effect ^b^ (+/−)**
A549/BALB-c	lung adenocarcinoma	1	+	n.e.
A549/CD-1	lung adenocarcinoma	3	+ (3x)	n.e.
HTB119/CD-1	small-cell lung carcinoma	2	+ (2x)	n.e.
HT1080/BALB-c	fibrosarcoma	2	+ (2x)	+
HT1080/CD-1	fibrosarcoma	37 ^c^	+ (29x)	+
M21/BALB-c	melanoma	6	+ (6x)	n.e.
M21/CD-1	melanoma	5	+ (4x)	n.e.
MCF-1/CD-1	breast carcinoma	1	+	n.e.
MDA-MB-435/BALB-c	melanoma	2	+ (2x)	+
SKBR3/CD-1	breast carcinoma	5	+ (4x)	+
U87/CD-1	glioblastoma	4	+ (4x)	+
**Syngeneic Mouse Tumor Model** **(Cell Line/Mouse Strain)**	**Histology** **(Murine)**	**No. of Experim.** **with ≥ 5 Mice/Group**	**Therapeutic** **Effect ^a^ (+/−)**	**Imaging** **Effect ^b^ (+/−)**
B-16/C57BL6	melanoma	2	+ (2x) ^d^	+ ^d^
LLC/C57BL6	lung carcinoma	1	(-)	n.e.

+ indicates experiments with significant (Mann–Whitney test) tumor growth retardation or regression by tTF-NGR in comparison to controls; −, no obvious therapeutic effects; n.e., not examined; ^a^, negative therapy results often occurred due to suboptimal test conditions like underdosage, tardy/late therapy effects, fast growing tumor entities etc.; ^b^, e.g., reduction of blood flow or vessel occlusion, observed by various imaging methods (contrast-enhanced ultrasound, CEUS; fluorescence reflectance imaging, FRI; magnetic resonance tomography, MRT; single photon emission computed tomography, SPECT); ^c^, three triple-combination experiments with Avastin, Doxorubicin, and tTF-NGR (1x +, 2x −) were excluded due to suboptimal study design; ^d^, inhibition of lung metastasis (clinically, then by imaging and post mortem examination).
